# Bone Metastases in Non-Seminomatous Germ Cell Tumors: A 20-Year Retrospective Analysis

**DOI:** 10.3390/jcm13113280

**Published:** 2024-06-02

**Authors:** Romane Gille, Benoît Allignet, Floriane Izarn, Patrice Peyrat, Helen Boyle, Aude Fléchon

**Affiliations:** 1 Campus Lyon Sud Charles Mérieux, University Claude-Bernard Lyon 1, 69921 Oullins-Pierre-Bénite, France; floriane.izarn@lyon.unicancer.fr; 2 Department of Medical Oncology, Centre Léon Bérard, 28 rue Laennec, 69673 Lyon Cedex, France; helen.boyle@lyon.unicancer.fr (H.B.); aude.flechon@lyon.unicancer.fr (A.F.); 3 Department of Radiation Oncology, Centre Léon Bérard, 28 rue Laennec, 69673 Lyon Cedex, France; benoit.allignet@lyon.unicancer.fr; 4 Univ Lyon, INSA-Lyon, Université Claude Bernard Lyon 1 , CNRS, Inserm, CREATIS UMR 5220, U1294, 69621 Lyon, France; 5 Department of Surgery, Centre Léon Bérard, 28 rue Laennec, 69673 Lyon Cedex, France; patrice.peyrat@lyon.unicancer.fr

**Keywords:** bone metastases, non-seminomatous germ cell tumor, testicular cancer, prognosis, chemotherapy

## Abstract

**Background:** Non-seminomatous germ cell tumors (NSGCTs) represent a rare yet the most prevalent malignancy among young men. Bone metastases (BMs) are exceedingly uncommon in this neoplasm, and available data regarding the initial disease presentation, survival outcomes, and prognostic significance of BMs are limited. **Methods:** We conducted a retrospective analysis of 40 NSGCT patients with BMs treated between 2001 and 2021 in our tertiary care center. The cohort was stratified into synchronous (n = 29) and metachronous (n = 11) groups based on the presence of BM at diagnosis or only at relapse, respectively. We assessed overall survival (OS), progression-free survival (PFS), disease presentation, and treatments. **Results:** After a median follow-up of 93 months, the 5-year PFS and OS rates were 37.6% and 53.9% in the synchronous group and 18.2% and 36.4% in the metachronous group, respectively. At the initial diagnosis, most patients were classified into the IGCCCG poor prognostic group (n = 34, 85%). BMs were mostly asymptomatic (n = 23, 57.5%), involved the spine (n = 37, 92.5%), and could become visible only after disease response (n = 4, 10%). A pathological examination of resected bone lesions after first-line treatment revealed necrosis (n = 5, 71.4%), teratoma, or seminoma (both n = 1, 14.3%). At first relapse, eight patients in the synchronous group did not experience bone recurrence, while eight patients experienced recurrence at the initial affected bone site. **Conclusions:** In NSGCT patients, BMs often present asymptomatically and may initially be unnoticed. However, these patients may have a poorer prognosis compared to those in the IGCCCG poor prognostic group. Further studies including control groups are needed to assess the independent prognostic significance of BMs.

## 1. Introduction

Germ cell tumors (GCTs) are a rare disease, with an incidence of 74,000 per year worldwide [1]. However, they are the most common neoplasia in men aged 20 to 40, and their incidence is increasing [1]. Several risk factors are well known, such as age, cryptorchidism, personal and familiar history of testicular GCT, genetic syndromes such as Down or Klinefelter, and the use of cannabis. More recently, endocrine disruptors have been described as risk factors [2]. 

Due to their high chemo-sensitivity, the prognosis of GCT remains excellent. Approximately 80% of patients are cured with a protocol involving surgery and cisplatin-based chemotherapy [3,4,5]. Even in cases of metastatic disease, the 5-year overall survival (OS) rate remains above 95% [6]. Nevertheless, prognosis within the metastatic group varies widely. The International Germ Cell Cancer Collaborative Group (IGCCCG) has categorized patients into three prognostic groups based on pathology, primary tumor site, tumor markers levels, and metastatic sites [6]. 

In non-seminomatous GCTs (NSGCTs), non-pulmonary visceral metastasis (NPVM) is an independent poor prognostic factor, resulting in a 5-year OS rate of only 67% [7]. While bone metastases (BMs) are common in many metastatic cancers, they are notably rare in GCTs [8]. Limited data from small studies suggest that the frequency of BM is around 3% at the initial diagnosis and 9% at relapse [9,10].

Due to the scarcity of comprehensive data, initial disease presentation, predictive factors, preferred imaging modalities, and the prognostic significance of BM in NSGCTs are unclear [7,11]. 

Currently, there is no consensus on the optimal management and treatment strategies for these patients, leading to potential variations in clinical practice.

Given this substantial gap in knowledge, our study aims to provide a detailed assessment of the characteristics, treatments, and survival outcomes of NSGCT patients presenting with BM. By addressing these critical areas, we hope to enhance the understanding and management of this rare but significant complication in NSGCTs.

## 2. Methods

### 2.1. Patients

We conducted a retrospective analysis of NSGCT patients who were treated in a tertiary care center at the Léon Bérard Cancer Center in Lyon, France, between 2001 and 2021. Inclusion criteria comprised male sex, age 18 years or older, treated for pathologically confirmed NSGCT, and the radiological or pathological confirmation of BM during their treatment course. 

Patients were divided into two groups: synchronous and metachronous disease. The synchronous group included patients diagnosed with BM on radiological exams between the initial work-up and the last chemotherapy (ChT) course of the first-line chemotherapy (ChT). The metachronous group included patients who developed BM after completing the last ChT course of the first-line treatment.

Personal data processing was conducted in accordance with the French Reference Methodology n°004 (MMR 004) of the National Informatic and Liberties Commission. Informed consent was obtained from all patients. The study protocol received approval from the Centre Léon Bérard institutional ethical and scientific review board. 

### 2.2. Outcomes

The primary outcome was OS, which was calculated from the initial diagnosis to death from any cause. Secondary endpoints included progression-free survival (PFS), the number and location of BM, and administered treatments. PFS was calculated from the initial diagnosis to any local or distant progression or death from any cause.

### 2.3. Statistical Analysis 

Categorical variables were reported as counts and percentages, and continuous variables were reported as means and ranges. Median follow-up duration was determined using the reverse Kaplan–Meier method. Survival rates were estimated using the Kaplan –Meier method and compared using the log-rank test. Two-sided *p*-values were considered significant if <0.05. Statistical analyses were performed using R software version 4.5.2 (R Foundation for Statistical Computing, Vienna, Austria). 

## 3. Results

### 3.1. Patients’ Characteristics

Between 2001 and 2021, 374 patients were treated for metastatic NSGCTs at the Léon Bérard Cancer Center. Among them, 190 (51%), 71 (19%) and 112 (30%) were classified as good, intermediate, and poor prognosis according to the IGCCCG classification. The corresponding 5-year OS rates were 96.1%, 87.8%, and 62.6%, respectively. 

Moreover, 40 patients met the inclusion criteria, with 29 (72.5%) classified in the synchronous group and 11 (27.5%) in the metachronous group. At last update of 5 March 2024, the median follow-up was 93.0 months (IQR 44.52–141.55). The patients’ main characteristics are summarized in Table 1. The median age at diagnosis was 32 years (range, 19–65). Only three patients (7.5%) had a mediastinal primary tumor site. A pathological analysis revealed mixed NSGCTs and pure NSGCTs in 31 (77.5%) and 7 cases (17.5%), respectively. Further details of pathological analysis are provided in Supplementary Tables S1–S3.

At the initial diagnosis, all patients presented with elevated serum tumor markers, and 34 (85%) were classified in the IGCCCG poor prognosis group (details in Table 1).

### 3.2. Diagnosis of Bone Metastases

In the synchronous group, most BMs were diagnosed during initial staging and in the absence of symptoms (n = 12, 58.6%). Among these cases, four were identified using non-routinely recommended imaging modalities (18FDG-PET, n = two; bone scan, n = one; and MRI, n = one). In five other asymptomatic patients (17.2%), BMs were not initially visible during staging and were subsequently detected upon reevaluation using computed tomography (CT) after two or four courses of ChT. The remaining 12 (41.4%) patients presented with symptoms (pain and/or fracture) leading to a radiological diagnosis of BM. 

In the metachronous group, bone relapse was diagnosed in six asymptomatic patients (54.5%) through systematic CT or 18FDG-PET, while the other five (45.5%) presented symptoms leading to further investigation. 

BM biopsies were performed in 12 patients, revealing yolk sac tumor (n = 7), adenocarcinomatous transformation (n = 2), seminoma (n = 1), embryonal carcinoma (n = 1), and angiosarcoma (n = 1). 

### 3.3. Metastatic Presentation

In the overall population, 10 patients (25%) presented uncommon NPVM locations, including intestinal, serous, splenic, adrenal, or intracardiac sites (details in Table 2). Three patients (10.3%) presented exclusively with BM at diagnosis. The majority of BMs involved the spine (n = 37, 92.5%) and pelvic bones (n = 8, 20%) (details in Table 3). Most BMs were described as osteolytic (n = 21, 52.5%), and only one was purely osteosclerotic. 

At the initial diagnosis, most patients of the metachronous group (n = 10, 90.1%) had visceral metastases (details in Table 2).

### 3.4. First-Line Treatment and Outcome

All patients received standard cisplatin-based ChT as their first-line treatment. Thirty-nine patients (97.5%) achieved a partial response, while 1 patient experienced progressive disease.

Within the synchronous group, 11 patients (37.9%) underwent a resection of residual masses (details in Supplementary Tables S1–S3). Ten patients underwent post-ChT retroperitoneal lymph node dissection (RPLND). One patient died due to immediate post-RPLND pneumopathy. Subsequently, seven patients underwent surgical resection of BM (vertebrectomy, n = six; hemipelvectomy, n = one). A pathological examination of the resected bones revealed necrosis in five cases (71.4%) and teratoma and seminoma each in one case (14.3%). 

In the metachronous group, eight patients (72.7%) underwent RPLND after first-line ChT. A pathological examination of RPNLD specimens revealed teratoma in five cases and viable cells in three cases (yolk sac tumor, n = one; embryonal carcinoma, n = two). Two patients (18.2%) underwent a complete resection of all metastatic sites.

### 3.5. Relapse 

In the synchronous group, 17 patients (58.6%) experienced relapse, all occurring within the first year, with a median time to relapse of 3 months (range, 0–11). Among them, eight patients (47.0%) did not present with a bone relapse but metastases in the brain (n = four), lung and/or lymph nodes (n = three), and liver (n = one). Eight of the nine bone relapses (88.9%) occurred in the initially affected site. The second-line treatment consisted of standard-dose chemotherapy (SDCT; n = 7, 41.2%) or high-dose chemotherapy with an autologous stem cell transplant (HDCT; n = 9, 52.9%) (details in Supplementary Table S2).

In the metachronous group, all patients presented with at least either lung or nodal metastases at the first relapse, and six (54.5%) had a single BM (details in Table 2). Three patients (27.3%) experienced relapse more than 2 years after the initial treatment. The second-line treatment consisted of SDCT (n = 8, 72.7%) or HDCT (n = 3, 27.3%). No objective response was observed beyond the third line (mean number of ChT lines, n = four).

### 3.6. Progression-Free and Overall Survival 

**Figure 1:**
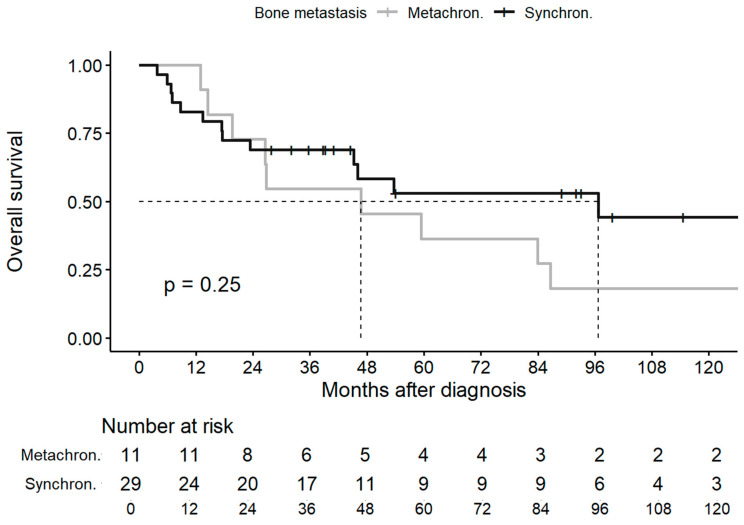
Overall survival in synchronous and metachronous groups. Abbreviations: metachron., metachronous; synchron., synchronous.

The median OS rates of the overall cohort, the synchronous group and the metachronous group were 59.4 months (95%CI, 45.2—not reached (NR)), 96.7 months (95%CI, 45.2—NR), and 46.7 months (95%CI, 26.5—NR), with 5-year OS rates of 47.6%, 53.1%, and 36.4%, respectively (Figure 1).

**Figure 2:**
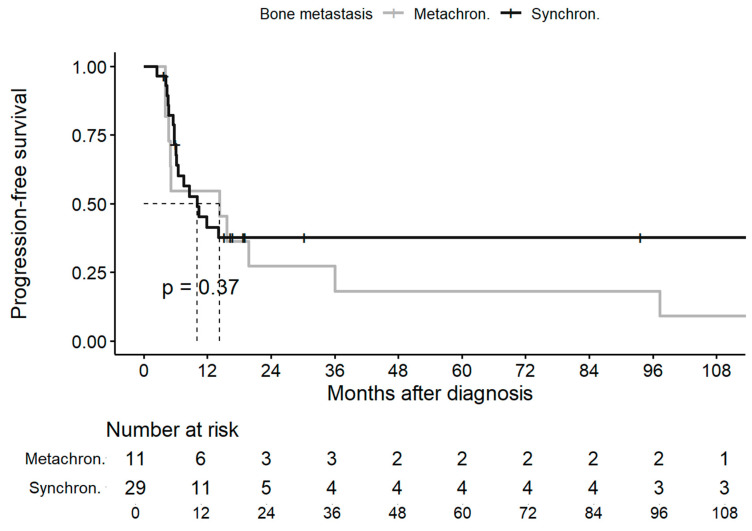
Progression-free survival in synchronous and metachronous groups. Abbreviations: metachron., metachronous; synchron., synchronous.

The median PFS rates of the overall cohort and the synchronous and metachronous groups were 10.4 months (95%CI, 6.2–97.3 months), 10.0 months (95%CI, 6.2—NR), and 14.3 months (95%CI, 5.0—NR), with 5-year PFS rates of 31.5%, 37.6%, and 18.2%, respectively (Figure 2).

### 3.7. Impact of BM Number and Location

**Figure 3:**
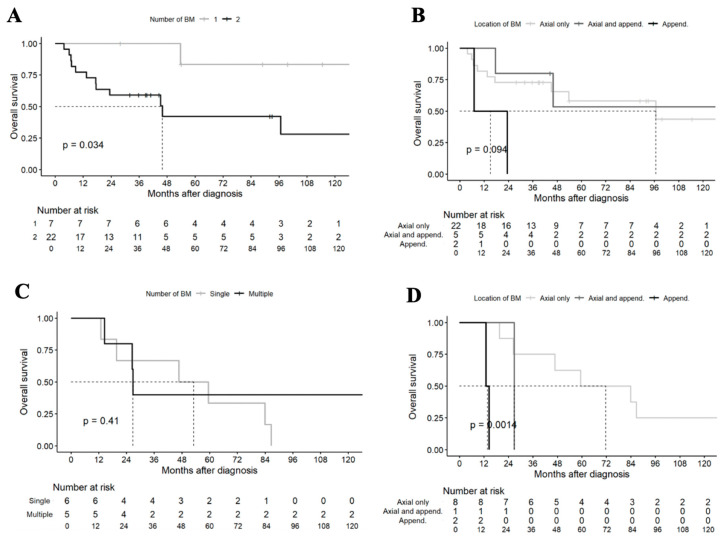
OS in patients by BM number and location. OS in synchronous group patients according to (**A**) number of BMs (unique or multiple) and (**B**) location (axial only, axial and appendicular, appendicular only). OS in metachronous group patients according to (**C**) number of BM (unique or multiple) and (**D**) location (axial only, axial and appendicular, appendicular only). Abbreviations: append., appendicular.

In the synchronous group (n = 29), patients with a single BM appeared to have better survival outcomes compared to patients with multiple BMs (median OS NR vs. 46 months; *p* = 0.034) (Figure 3A). Patients with BM limited to the appendicular skeleton (see details in Table 3) seemed to have a worse OS, although the results did not reached statistical significance (*p* = 0.094) (Figure 3B).

In the metachronous group (n = 11), the number of BMs did not show a significant impact on survival (Figure 3C). However, patients with appendicular BM had a significantly worse prognosis, whether or not associated with axial skeleton BM (*p* = 0.0096) (Figure 3D). 

## 4. Discussion

In NSGCT patients, BMs are uncommon and seem to carry a poor prognosis. In synchronous or metachronous BMs, the 5-year PFS rate was 37.6% and 18.2%, and the 5-year OS rate was 53.1% and 36.4%, respectively. Our results are consistent with those of the largest international study on synchronous BM in GCTs by Oing et al. [10]. In the NSGCT subgroup, they reported 2-year PFS and OS rates of 24% and 36%, respectively. These results contrast with the promising survival rates reported by the IGCCCG—Update Consortium in 2021 [7]. The Consortium included 9728 metastatic NSGCT patients treated between 1990 and 2013 and reported 5-year PFS and OS of 54% and 67% in the poor prognosis group, regardless of the site of NPVM. However, they did not specifically evaluate the prognostic significance of BMs nor reported their prevalence.

Our results suggest that metachronous BMs are associated with a poorer prognosis compared to synchronous BMs. However, a recent study conducted on 99 patients in the United States did not find any prognostic impact, although it did report similar results to ours about the prognosis of NSGCTs with BM [12]. Indeed, the outcomes of these patients were worse than expected based on the IGCCCG Consortium, with a 5-year PFS rate of 14.6% (95%CI 6.9–25.1) and a 5-year OS of 52.2% (95%CI 38.4–64.2).

Despite the potentially poorer prognosis associated with BMs, their presence at the initial diagnosis did not appear to impair the response to first-line ChT, with an objective response rate of over 95% in our study. In addition, necrosis without viable cells was found in most resected bones, suggesting that chemotherapy is also effective in this metastatic site. Therefore, BM is likely to reflect the aggressiveness of the disease rather than serve as an independent prognostic factor. 

Most BMs affected spine (92.5%) and pelvic bones (20%), which was consistent with previous publications [9,10,13,14]. As expected, the most frequent concurrent metastatic sites were lungs and lymph nodes. In contrast, a quarter of our patients presented with atypical metastatic sites such as the bowel, spleen, adrenal gland, or even intracardiac locations. Hence, physicians should pay attention to bone invasion when unusual metastases are seen and may consider additional 18FDG-PET to comprehensively investigate the extent of metastatic spread. Notably, we reported three patients with BM and no visceral metastases at initial diagnosis, which is rarely described in the literature [15,16,17,18,19]. 

Predictive factors for the development of BM in NSGCT patients are currently unknown. Oechsle et al. reported that patients with mediastinal primary NSGCTs, a histology of yolk sac tumors, or liver metastases were more likely to present initial BM [13]. In our study, pure yolk sac tumor histology was the most frequent pathological finding, and 80% of patients had elevated AFP levels at the initial diagnosis, which is in favor of the presence of a yolk sac or embryonal carcinoma component. 

The post-ChT resection of any residual mass measuring more than 1 cm is recommended by European and French guidelines, since approximately 40% of post-ChT cases reveal mature teratoma and 10% viable GCT [3,4,5,20,21]. In contrast, the role of bone surgery remains to be evaluated. Firstly, the necessity of this surgery is debatable since some patients who underwent RPLND without bone surgery did not experience a relapse in our study. Secondly, these particularly invasive procedures require an experienced orthopedic surgeon. Limited data are available regarding bone resection but suggest its feasibility and safety [22,23]. 

We observed dissimilar pathological findings between RPLND and BM, as already reported between thoracic surgeries and RPLND [24,25]. Thus, pathological evaluations of post-ChT RPLND may not reliably predict BM response. In addition, no recurrence was observed if no viable cells were found in a BM specimen, suggesting a potentially predictive value. Finally, 88.9% of bone relapses occurred in the initial location, supporting a therapeutic benefit of bone resection if indicated and safely feasible. 

At relapse, some studies suggest that HDCT could improve prognosis compared to SDCT. Oing et al. reported a significantly higher overall response rate to salvage HDCT (81% vs. 43%, *p* < 0.001) [15]. In our study, more than half of the patients of the synchronous group received HDCT as a second-line treatment. Due to our small sample size, statistical power was insufficient to analyze HDCT’s prognostic value. An EORTC randomized phase III trial is ongoing to compare SDCT and HDCT in relapsed or refractory GCT (TIGER Trial, NCT02375204, source: https://www.clinicaltrials.gov/study/NCT02375204?cond=NCT02375204&rank=1).

In our cohort, five (17.2%) asymptomatic patients who did not exhibit visible BM at diagnosis were found to have a ChT-induced osteoblastic reaction mimicking progressive disease, a phenomenon previously described in the literature [26,27]. Additionally, five other patients had BMs that were not initially detected upon staging CT but were later identified by nuclear imaging. Moreover, 18FDG-PET might be considered at the initial staging and/or after ChT completion when considering multifocal surgeries. Nevertheless, to the best of our knowledge, its diagnostic performance has never been compared to the chest–abdomen and pelvis CT in this indication. While a systematic screening for BM is not recommended in asymptomatic patients due to its rarity, there is a risk of missing initial metastatic locations, potentially leading to a misclassification of prognosis [9,10]. Other radiological innovations, such as dual-energy CT, may enhance the diagnosis and evaluation of BM [28]. By scanning at two different energies, this technique provides additional information about tissue composition. A publication reported that the combination of CT and water—hydroxyapatite images significantly improves the detection of BM [29]. Additionally, advancements in biological innovations such as liquid biopsies and micro RNAs may also enhance the diagnosis and follow-up of BM [30].

Our study has limitations, including its retrospective and monocentric design, which may introduce institutional bias. The lack of standardized guidelines for the management of BM in NSGCTs and the inherent diagnostic challenges contribute to a potential selection bias. Notably, 30% of our patients had a poor prognosis compared to 23.7% in the IGCCCG Update Consortium cohort, suggesting our sample may represent more advanced cases, especially treated in our tertiary care center [7]. Despite these limitations, our prognostic outcomes align with IGCCCG results, supporting the hypothesis of worse prognosis in the presence of BM [31,32]. Further prospective multicentric studies are needed to confirm our findings. For instance, patients with BM might benefit from treatment intensification trials. Furthermore, it could be interesting to stratify future intensification studies based on the presence of BM. This approach could help tailor more effective treatment strategies and potentially improve outcomes for this specific patient subgroup.

## 5. Conclusions

In NSGCT patients, BMs are often asymptomatic and may initially be unnoticed. However, these patients may have a poorer prognosis compared to those in the IGCCCG poor prognostic group. Further studies including control groups are needed to assess the independent prognostic significance of BM. 

## Figures and Tables

**Table 1:** Patient characteristics at initial diagnosis.

Characteristics	Overall (n = 40)	Synchronous (n = 29)	Metachronous (n = 11)
Median age (range)	32 (19–65)	32 (19–65)	29 (19–52)
Primary tumor site No (%)			
Testis	37 (92.5)	27 (93.1)	10 (90.9)
Mediastinum	3 (7.5)	2 (6.9)	1 (9.1)
Histopathology No (%)			
Mixed NSGCT	31 (77.5)	22 (75.8)	9 (81.2)
Pure NSGCT	7 (17.5)	5 (17.2)	2 (18.2)
Yolk sac tumor	4	3	1
Choriocarcinoma	1	1	0
Teratoma	2	1	1
Embryonal carcinoma	0	0	0
NA	2 (5)	2 (6.9)	0 (0)
IGCCCG prognosis group			
Good	1 (2.5)	0 (0)	1 (9)
Intermediate	5 (12.5)	0 (0)	5 (45.4)
Poor	34 (85)	29 (100)	5 (45.4)
Serum tumor markers level			
AFP elevated	33 (82.5)	25 (86.2)	8 (72.7)
<1000	10	8	2
1000–10,000	13	10	3
>10,000	9	6	3
NA	1	1	
hCG elevated	21 (52.5)	16 (55.2)	5 (45.4)
<5000	12	10	2
5000–50,000	3	1	2
>50,000	6	5	1
LDH elevated	30 (75)	22 (75.9)	8 (88.9)
<1.5 N	2	2	0
1.5 N–10 N	24	16	8
>10 N	4	4	0
NA	5		
TNM stage ^1^	n = 37	n = 27	n = 10
T			
Tx	8 (21.6)	7 (26)	1 (10)
T1	14 (37.8)	7 (26)	7 (70)
T2	7 (18.9)	5 (18.5)	2 (20)
T3	8 (21.6)	8 (29.6)	0 (0)
T4	0 (0)	0 (0)	0 (0)
N			
N0	9 (24.3)	7 (26)	2 (20)
N1	4 (10.8)	4 (14.8)	0 (0)
N2	6 (16.2)	4 (14.8)	2 (20)
N3	18 (48.6)	12 (44.4)	6 (60)
M			
M0	1 (2.7)	0 (0)	1 (10)
M1	36 (97.3)	27 (100)	9 (90)
M1a	5	0	5
M1b (NPVM)	31	27	4

^1^ for testicular tumors only. Abbreviations: IGCCCG, International Germ Cell Cancer Collaborative Group; NPVM, non-pulmonary visceral metastasis; NSGCT, non-seminomatous germ cell tumor.

**Table 2:** Visceral metastases at diagnosis in synchronous and metachronous group.

Location Number (%)	Synchronous Group (n = 29)	Metachronous Group (n = 11)
Lymph node	22 (75.9)	8 (72.7)
Lung	18 (62.1)	8 (72.7)
NPVM	23 (82.1)	5 (27.3)
Liver	11 (37.9)	3 (27.3)
Brain	4 (13.8)	0 (0)
Pleura	1 (3.4)	0 (0)
Muscle	2 (6.9)	0 (0)
Peritoneum	1 (3.4)	0 (0)
Cardiac	0 (0)	1 (9.1)
Spleen	1 (3.4)	1 (9.1)
Duodenum	1 (3.4)	0 (0)
Adrenal gland	2 (6.9)	0 (0)
None	3 (10.3)	1 (9.1)

Abbreviations: NPVM, non-pulmonary visceral metastasis.

**Table 3:** Bone metastases in synchronous and metachronous group.

Bone Metastases Number (%)	Synchronous Group (n = 29)	Metachronous Group (n = 11)
Number of BM		
1	7 (24.1)	6 (54.5)
>1	22 (75.9)	5 (45.5)
Location: axial skeleton		
Spine	26 (89.7)	11 (100)
Ribs	4 (13.8)	2 (18.2)
Sternum	1 (3.4)	0 (0)
Location: appendicular skeleton		
Pelvic bones	7 (24.1)	1 (9)
Femur	1 (3.4)	2 (18.2)
Humerus	0 (0)	3 (27.3)
Type		
Osteolytic	15 (51.7)	6 (54.5)
Osteoblastic	0 (0)	1 (9)
Mixed	3 (10.3)	2 (18.2)
Undescribed before systemic treatment	11 (37.9)	2 (18.2)

## Data Availability

The raw data supporting the conclusions of this article will be made available by the authors on request.

## Supplementary Materials

The following supporting information can be downloaded at: https://www.mdpi.com/article/10.3390/jcm13113280/s1, Table S1: Details of primary tumor site’s histological pattern; Table S2: Histology of residual masses in the synchronous group (n = 11); Table S3: Second line chemotherapy at relapse in the synchronous group.
