# Supplementary material for: Bone Metastases in Non-Seminomatous Germ Cell Tumors: A 20-Year Retrospective Analysis
Source: J Clin Med. 2024 Jun 2;13(11):3280. doi: 10.3390/jcm13113280 (PMC11172778; doi:10.3390/jcm13113280)
Supplement: Supplementary file 1 [file jcm-13-03280-s001.zip › jcm-2966430-supplementary.pdf]

SUPPLEMENTARY MATERIAL

Table S1. Details of primary tumor site's histological pattern.

Characteristics	All (n = 40)	Synchronous (n = 29)	Metachronous (n = 11)
Primary tumor site No (%)			
Testis	37 (92.5)	27 (93.1)	10 (90.9)
Mediastinum	3 (7.5)	2 (6.9)	1 (9.1)
Histopathology No(%)			
Mixed NSGCT	31 (77.5)	22 (75.8)	9 (81.2)
Pure NSGCT	7 (17.5)	5 (17.2)	2 (18.2)
NA	2 (5)	2 (6.9)	0 (0)
Pure tumors histology No(%)	n = 7	n = 5	n = 2
Yolk sac tumor	4 (57.1)	3 (60)	1 (50)
Choriocarcinoma	1 (14.3)	1 (20)	0 (0)
Teratoma	1 (14.3)	1 (20)	0 (0)
Embryonal carcinoma	1 (14.3)	0 (0)	1 (50)
Mixed tumors histology No (%)	n = 31	n = 22	n =9
Yolk sac tumor	18 (58)	16 (72.7)	6 (66.7)
Choriocarcinoma	5 (16.1)	4 (18.2)	1 (11.1)
Teratoma	19 (61.3)	12 (54.5)	7 (77.8)
Embryonal carcinoma	18 (58)	11 (50)	7 (77.8)
Seminoma	12 (38.7)	10 (45.4)	2 (22.2)
Lympho-vascular embols No (%)			
Yes	15 (37.5)	13 (44.8)	2 (18.2)
No	14 (35)	7 (24.1)	7 (63.6)
NA	11 (27.5)	9 (31)	2 (18.2)

Table S2. Histology of residual masses in the synchronous group (n=11).

Patient	Histology of RPLND	Bone surgery	Histology of BM	Relapse
3	Necrosis, Teratoma	No	-	Yes
11	Necrosis	Yes	Necrosis	No
15	Necrosis	Yes	Necrosis	No
16	Necrosis	Yes	Necrosis	No
18	Necrosis, Teratoma	Yes	Teratoma	Yes
21	Necrosis	Yes	Seminoma	Yes
25	Necrosis	No	-	No
27	Necrosis	Yes	Necrosis	No[1]
31[2]	Teratoma[2]	Yes	Necrosis	No
34	Teratoma	No	-	No
43	Necrosis	No	-	No

[1]died of immediate post-operative complications

[2]primary mediastinal tumor

Table S3. Second line chemotherapy at relapse in the synchronous group.

Second line chemotherapy	Number of patients (n = 16[1])	Best treatment response	Progression after second line
SDCT	7		
TIP	4	4 RPm-	3 / 4
VeIP	1	1 RPm-	1 / 1
GIP	1	1 RPm-	1 / 1
PAC	1	1 RPm+	1 / 1
HDCT	9	2 PD	2 / 2
		1 RPm+	1 / 1
		6 RPm-	2 / 6

[1]one decease before second line treatment. Abbreviations: SDCT. standard dose chemotherapy; HDCT. high dose chemotherapy with autologous stem cell transplant; TIP. paclitaxel ifosfamide cisplatin; VeIP. vinblastine ifosfamide cisplatin; GIP. gemcitabine ifosfamide cisplatin; PAC. cisplatin adriamycin cyclophosphamide; POMB ACE. cisplatin vincristine methotrexate bleomycin actinomycin D cyclophosphamide etoposide; RPm-. partial response negative markers; RPm+. partial response positive markers; SD. stable disease; PD. progressive disease.
